# Possibility of using the yes/no Angoff method as a substitute for the percent Angoff method for estimating the cutoff score of the Korean Medical Licensing Examination: a simulation study

**DOI:** 10.3352/jeehp.2022.19.23

**Published:** 2022-08-31

**Authors:** Janghee Park

**Affiliations:** Department of Medical Education, Soonchunhyang University College of Medicine, Cheonan, Korea; Hallym University, Korea

**Keywords:** Educational measurement, Probability, Reproducibility of results, Republic of Korea

## Abstract

**Purpose:**

The percent Angoff (PA) method has been recommended as a reliable method to set the cutoff score instead of a fixed cut point of 60% in the Korean Medical Licensing Examination (KMLE). The yes/no Angoff (YNA) method, which is easy for panelists to judge, can be considered as an alternative because the KMLE has many items to evaluate. This study aimed to compare the cutoff score and the reliability depending on whether the PA or the YNA standard-setting method was used in the KMLE.

**Methods:**

The materials were the open-access PA data of the KMLE. The PA data were converted to YNA data in 5 categories, in which the probabilities for a “yes” decision by panelists were 50%, 60%, 70%, 80%, and 90%. SPSS for descriptive analysis and G-string for generalizability theory were used to present the results.

**Results:**

The PA method and the YNA method counting 60% as “yes,” estimated similar cutoff scores. Those cutoff scores were deemed acceptable based on the results of the Hofstee method. The highest reliability coefficients estimated by the generalizability test were from the PA method and the YNA method, with probabilities of 70%, 80%, 60%, and 50% for deciding “yes,” in descending order. The panelist’s specialty was the main cause of the error variance. The error size was similar regardless of the standard-setting method.

**Conclusion:**

The above results showed that the PA method was more reliable than the YNA method in estimating the cutoff score of the KMLE. However, the YNA method with a 60% probability for deciding “yes” also can be used as a substitute for the PA method in estimating the cutoff score of the KMLE.

## Introduction

### Background/rationale

The standard-setting method has been introduced not only for nationwide high-stakes examinations [1], but also for single school-based examinations [2] in Korea. The percent Angoff (PA) method (modified Angoff method) is the oldest and most popular standard-setting method. In the PA method, panelists make judgments about each item to estimate the cutoff score [3]. There are 2 difficulties with most panels during the Angoff standard-setting process: the first is determining a minimally competent examinee (MCE), and the second is estimating the MCE’s correct percentage [4]. To reach a consensus about the MCE, panelists have a long discussion before judging the items.

The yes/no Angoff (YNA) method has been used as a variation of the Angoff method. The YNA asks panelists to decide whether the MCE chooses a correct or incorrect answer for each item, instead of the percent correct for each item; thus, it is an easier cognitive task than estimating probabilities [4].

The YNA has consistently produced a higher cutoff score than the Ebel method has, but it offers the possibility of faster and easier standard-setting exercises for local, minimum-stakes performance exams [5]. The YNA method forces the panelists to decide “yes” or “no,” which introduces a systematic bias that could produce a substantially distorted cutoff score.

The Korean Medical Licensing Examination (KMLE) has a large volume of items—360 items in 2017 and 320 items in 2022—most of which involve problem-solving, and more than 30% of the items have shown a correct answer rate of 90% or higher. Ahn et al. [6] suggested that the conventional standard-setting process, in which all panelists should decide on all items, was not effective for panels requiring considerable time and effort. Furthermore, standard-setting for the mock Korean Nursing Licensing Examination by the Angoff method was reported to be applicable. The item number for the mock exam was 295, so the item rating was done by 4 groups of 16 raters after dividing all the items into 4 groups [1]. To reduce the panel’s burden in deciding on a large volume of items of a high cognitive level for a high-stakes test, standard-setting with subtests made by stratified item sampling was recommended [7,8]. The YNA method is another idea to reduce the panel’s burden in the standard-setting process; thus, it is essential to compare the cutoff score and reliability between the PA and the YNA.

It is necessary to compare the reliability in terms beyond the cutoff score between various standard-setting methods. The less error, the higher the reliability. To find the cause of the error, an analysis based on generalizability theory is usually used. Generalizability theory [9] has been commonly applied to quantify the relative influences of each factor (e.g., panelists, items, rating rounds) on the variability (reliability coefficient) of cutoff scores, standard error (SE) of measurement, and panelist agreement [10].

### Objectives

This study aimed to compare the results of the PA method and the YNA method for estimating the cutoff score of the KMLE. Specifically, cutoff scores, reliability coefficients, and the error sources and variances were compared between these 2 methods.

## Methods

### Ethics statement

The panelist rating data in this study were reused from open access data from Harvard Dataverse. The data were produced as a result of research approved by the institutional review board (IRB approval no., 202001-SB-003) for a study wherein Park et al. [7] in 2020 examined the similarity of the cutoff score in the KMLE 2017 test sets with different item amounts using the modified Angoff, modified Ebel, and Hofstee standard-setting methods for the KMLE. Therefore, neither further approval by the IRB nor obtainment of informed consent was required.

### Study design

This is a simulation study to compare 2 standard-setting methods using open-access KMLE 2017 panelist data [7].

### Setting

In the original research on the open-access data reused in this study, full-day standard-setting workshops were held on February 8 and 22, 2020. Due to the coronavirus disease 2019 pandemic, the workshop was conducted in person on the first day and online on the second day. On the first day, the panelists had an orientation to setting scores using the Angoff method.

### Participants

Table 1 shows the 15 panelists’ characteristics They were recruited by the authors of the previous article [7]. Their specialties were divided into the following 4 categories. Most of the experts had at least 3 years of experience in item development for licensing examinations.

### Variables

Cutoff scores, reliability coefficients, and the error sources and variances were included as test variables.

### Measurement/data sources

The panel’s decision regarding the PA method from the original 2017 data was used. The YNA data were calculated from the original PA data for the various versions. The open-access data was obtained from Harvard Dataverse (https://doi.org/10.7910/DVN/CBOUPU) (Dataset 1). In the open-access data, 15 panelists set the cutoff score for 360 items of the KMLE in 2017. The percent and yes/no Angoff data are available from Dataset 2. According to the KMLE test report, 3,263 examinees participated, and 3,259 applicant scores were analyzed. Except for 4 droppers, the passing rate was 92.8% (3,105 passed and 158 failed). The mean difficulty was 72.1%, the mean discrimination was 0.17, the reliability was 0.926, and the standard deviation (SD) was 7.33 [10].

#### Percent Angoff and yes/no Angoff method

Data estimated by all panelists with the estimated correct percent of the MCE according to the PA method were used. In the YNA method, panelists should decide whether the MCE chooses a correct or false answer for each item. It is commonly imagined that the probability of deciding “yes” is 50%, but in this study, we had 5 probabilities of deciding or categorizing “yes”: 50%, 60%, 70%, 80%, and 90% (Table 2). Although 60% was the cutoff score of the KMLE, 70%, 80%, and 90% were calculated to identify the overall pattern.

#### Cutoff score

The cutoff scores were compared between the PA and YNA methods, in which the criteria to decide yes or no were 50%, 60%, 70%, 80%, and 90%. There were 4 ways to calculate the cutoff score in this study: first, the mean score of the panel’s decision (M); second, the mean score minus the standard deviation of the panel’s decision (M-SD); third, the mean score minus the standard error of the panel’s decision (M-SE); and fourth, the mean score minus the standard error of measurement (SEM) (M-SEM). The standard-setting method has a classification error, which includes false-positive and false-negative classifications; thus, it would be reasonable to modify the cutoff score. The cutoff score has an error, so we can calculate the modified cutoff score by considering the SE. Modifying the cutoff score with the SE of measurement (SEM) is another way to correct the error. The SE focuses on the panel’s decision, whereas the SEM emphasizes the quality of the test and its reliability [2,4]. The calculation formulas are as follows:


$$\begin{array}{l} SE\;=\;s_{x}/\sqrt{n}\\ s_{x}:\;\text{SD of observed score x; n: number of samples}\\ SEM\;=\;SD\sqrt{1-rxx}\\ \text{SD:}\;\text{the SD of test scores; rxx: the reliability of the test} \end{array}$$

#### Generalizability test

The effect size of the error variance can be estimated by the generalizability test [9], as well as the generalizability coefficient and the dependability coefficient. The used generalizability theory model was a random facet nested design, symbolized as [i×(s:p)], in which item (i) is crossed with panelist (p), and panelist is nested in specialty (s). There were 360 items, 15 panelists, and 4 specialty categories. The data entry is shown in Table 3.

#### Hofstee method

Ahn et al. [6] in 2018 proposed a 2-step standard-setting process for deciding the final cutoff score. The first step involves setting the standard with the modified Angoff or Ebel method and checking whether these cutoff scores developed in the first step are acceptable with the Hofstee method [6]. Park et al. [7] in 2020 used a standard-setting with the Hofstee method in KMLE in 2017. In the Hofstee method, panelists should answer 4 questions comprising minimum and maximum acceptable passing scores and failure rates [7]. The acceptable failure rates for the panelists were 10.2% (maximum) and 4.0% (minimum). The acceptable cutoff scores were 69.5% (highest) and 56.25% (lowest). The calculated cutoff score was 61.9% for the 2017 KMLE on a percentile scale. The cutoff scores of this study will be checked for acceptability with a Hofstee graph for the KMLE [7].

### Bias

None.

### Study size

This study was based on the panelists’ opinions, so the study size was not estimated.

### Statistical methods

A descriptive analysis was done using IBM SPSS ver. 27.0 (IBM Corp., Armonk, NY, USA). G string V was used for the analysis based on generalizability theory [11].

## Results

### Comparison of cutoff scores between the PA method and the YNA method

The results of the standard-setting process using the PA and YNA methods are presented in Table 4. The cutoff scores were compared between the PA method and YNA method, in which the criteria to decide “yes” or “no” were 50% (YNA-50%), 60% (YNA-60%), 70% (YNA-70%), 80% (YNA-80%), and 90% (YNA-90%). There were 4 ways to calculate the cutoff score in this study: first, the M; second, the M-SD; third, the M-SE; and fourth, the M-SEM. The SD and reliability of the KMLE test scores were 7.33 and 0.926, respectively, so the calculated SEM was 1.99. The SE of measurement was 1.99 [12].

**Fig. 1.**
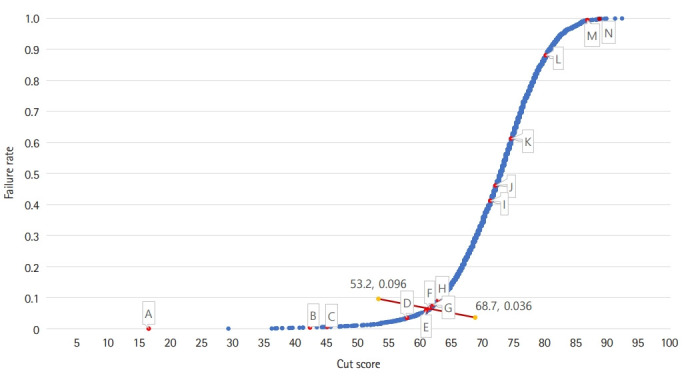
Calculated cutoff scores and Hofstee graph for the Korean Medical Licensing Examination in 2017. The original Hofstee graph cited from Park et al. [7] in 2020. A: YNA-90% (M-SEM), YNA-90% (M-SD), YNA-90% (M-SE), YNA-90% (M), YNA-80% (M-SD); B: YNA-80% (M-SE), YNA-80% (M-SEM), YNA-80% (M), YNA-70% (M-SD); C: YNA-70% (M-SE); D: YNA-70% (M-SEM), YNA-70% (M); E: PA (M-SD); F: YNA-60% (M-SD), PA (M-SEM); G: PA (M-SE); H: PA (M); I: YNA-60% (M-SE); J: YNA-60% (M-SEM); K: YNA-60% (M); L: YNA-50% (M-SD); M: YNA-50% (M-SE), YNA-50% (M-SEM); and N: YNA-50% (M). YNA, yes/no Angoff; PA, percent Angoff; SD, standard deviation; M, mean; SEM, standard error of measurement; SE, standard error.

According to the Hofstee results in Fig. 1, the maximum acceptable cutoff score was 69.5%, the minimum acceptable cutoff score was 56.25%, and the final estimated cutoff score was 61.9% for the 2017 KMLE [10]. The cutoff scores with the PA, YNA with 60% (YNA-60), and with M-SD, were acceptable based on the Hofstee results. Although the cutoff scores were above 69.5% (the maximum acceptable cutoff score), most of the cutoff scores with the YNA 60% method were nearest and most similar to the Hofstee results.

In Fig. 1, the lowest cutoff score and failure rate were marked with A, B, and C. The cutoff scores marked with D, E, F, and G were within the Hofstee score range. The highest cutoff score and failure rate were marked with I, J, K, L, M, and N. With A, we marked YNA-90% (M-SEM), YNA-90% (M-SD), YNA-90% (M-SE), YNA-90% (M), and YNA-80% (M-SD); with B, YNA-805 (M-SE), YNA-80% (M-SEM), YNA-80% (M), YNA-70% (M-SD); and with C, YNA-70% (M-SE). With D, we marked YNA-70% (M-SEM) and YNA-70% (M), whereas PA (M-SD) was marked with E, YNA-60% (M-SD) and PA (M-SEM) were marked with F, PA (M-SE) was marked with G, and PA (M) was marked with H. YNA-60% (M-SE) was marked with I, YNA-60% (M-SEM) with J, YNA-60% (M) with K, YNA-50% (M-SD) with L, YNA-50% (M-SE) and YNA-50% (M-SEM) with M, and YNA-50% (M) with N.

### Comparison of the size of error source between the PA and YNA methods

**Fig. 2.**
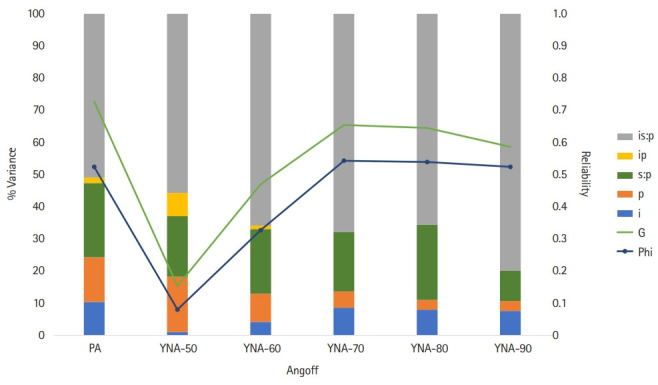
The variance percent and reliability between the percent Angoff (PA) and yes/no Angoff (YNA) methods. I, item; P, panelist; S, specialty.

The estimated variance component of the [i×(s:p)] model was analyzed using the generalizability test. The results are presented in Table 5 and Fig. 2.

Compared to PA, YNA-50% had a smaller item-related error (i), but a larger rater-item interaction (ip). Although the effect size of the panelist (p) was increased, the variance according to the specialty of the panelist (s:p) was similar. In YNA-50%, the size of the unexplained variance increased, which lowered the reliability significantly. In other words, YNA-50% reduced the differences between items, but allowed the panelists to rate each item differently.

Comparing the YNA method, YNA-50% and YNA-60% had a relatively small item-related variance (i), but the interaction between the panelist and item (ip) was large. If the probability used in the YNA method was more than 70%, the item variance (i) was similar, and there was little interaction between the item and the panelist (ip).

Comparing the variance related to the panelists, the difference between each specialty (s:p) was similar in each method, but the difference between the individual characteristics of the panelist (p) according to the method was substantial.

**Fig. 3.**
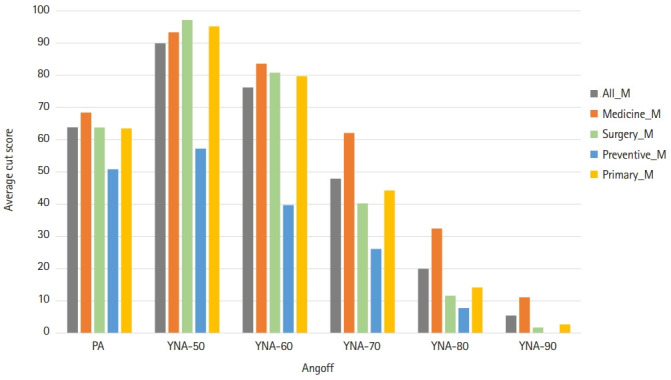
The average cutoff scores by specialty category between the percent Angoff (PA) and yes/no Angoff (YNA) methods. M, mean.

The reliabilities of YNA-70%, YNA-80%, and YNA-90% were similar, and it could be inferred that the average of the PA was 63.5 (Table 4). It can be said that the panelists evaluated items similarly when the probability was more than 70%. The average between specialty groups was calculated to analyze why the variance sizes of specialty were similar (Fig. 3). Overall, a similar pattern was found for each specialty regardless of the method, but 70% to 90% of internal medicine specialists evaluated the correct answer ratio as higher than other specialties.

## Discussion

### Key results

According to the Hofstee's results [7], the cutoff scores of PA were acceptable, being 63.5% (mean), 58% (M-SD), 62.1% (M-SE), and 61.5% (M-SEM). Among the cutoff scores of the YNA method, the cutoff score (61.4) calculated as M-SD with YNA-60% was acceptable and similar to the cutoff scores of PA. The effect size of error variance was estimated with generalizability theory. Compared to PA, YNA-50% had a smaller item-related error (i), but a larger panel-item interaction (ip). Although the effect size of the panelist (p) was increased, the variance according to the specialty of the panelist (s:p) was similar. Comparing the variance related to the panelist, the difference between each specialty (s:p) was similar by standard setting method, but the difference between the individual characteristics of the panelist (p) by method was large. The average cutoff score between specialty groups showed a similar pattern for each specialty regardless of the method; however, 70-90% Y/N Angoff of internal medicine of internal medicine specialists evaluated the correct answer ratio as higher than other specialties. The highest reliability coefficients estimated with generalizability theory were found for the PA, YNA with correct percentages of 70%, 80%, 60%, and 50% for the MCE to choose yes or no.

### Interpretation

When selecting an acceptable cutoff score based on the Hofstee method result, the PA and YNA-60% (mean score minus the SD) methods were most appropriate. The Hofstee method serves as a guideline for selecting an appropriate cutoff score because it shows the maximum and minimum passing scores and failure rates agreed upon by the panelists [6,7]. When the error variances were analyzed with generalizability theory, PA had higher reliability than YNA. This means that PA had higher intra- and inter-panelist consistency than YNA. In the YNA method, the reliability coefficient according to the percentage of guesses that choose “yes” increased from 50% to 70% but decreased from 80% to 90%. When the panelists set the probability of “yes” to 70%, the panelists’ consistency was estimated to be relatively high. The panelist’s specialty was the main cause of the error, and the variance size of the panel’s specialty was similar regardless of the setting method.

### Comparison with previous studies

This study compared the probabilities of correct answers for a person with minimum competency between PA and YNA methods. Similar to previous studies [10,11], the reliability coefficients of PA were higher than those of YNA, and the cutoff scores of YNA were higher than those of PA, so the failure rate was also higher. The YNA method is faster and easier to decide for panelists, but the reliability is relatively lower, so the YNA is good for use in local, medium-stakes performance exams. [5].

According to the results of the generalizability test in this study, the magnitude of the panelists’ variance was large, and the panelist’s specialty had a great deal of influence on the score on the written test. Because most results of generalizability were analyzed with a performance test, the variance of the professor’s specialty for a clinical performance examination with a single rater, such as a standardized patient or a professor, could not be calculated [10].

### Limitations

This study compared the PA and YNA methods. The YNA data were hypothetical data calculated from the PA data, and the results between simulation data and real data were compared.

### Suggestions

Although the error variance size of the panelists was high, there are not many papers on the cause of the error in panels. The panelist’s specialty was explained as a major error source. Because this study found that the different specialties of the panelists were a source of systematic error, it will be possible to analyze the rating pattern of the panelist in the future, implement pre-training to reduce errors between panelists, and adjust the score after determining the cutoff score. The YNA method was suggested as an alternative to reduce the burden on panelists. When there are many items to be decided by the panelists, sub-tests among all items have also been suggested as an alternative [7,8]. In order to reduce the burden of panelists and increase reliability when setting standards, an alternative can be to adopt various methods such as sub-tests and simplified methods.

### Generalizability

Although it is essential to set a reliable cutoff score in the criterion-referenced evaluation, standard-setting is a time-consuming process that is burdensome for panelists. The PA method is popular but not easy to use to determine the probabilities of many items. It is reasonable to determine the criteria for “yes” or “no” with a 60% chance for local and medium-stakes tests to reduce time and provide an easy process for panelists.

### Conclusion

The PA method is more reliable than the YNA method. If the YNA method is used, the 60% criterion to decide “yes” by the panelist is recommended because it had a more reliable coefficient, acceptable scores based on Hofstee results, and a similar cutoff score to the PA method. The panelist’s specialty was the main source of error variance, and the size of the ratio was similar regardless of the setting methods.

## Figures and Tables

**Table 1.** Characteristics of panel members at medical schools in Korea [9]

Category	Content	No. (%)
Panelists’ specialties	- Medicine: internal medicine (2 panelists), pediatrics (2 panelists), psychiatry (2 panelists)	15 (100.0)
	- Surgery: surgery (2 panelists), obstetrics & gynecology (2 panelists)	
	- Law: preventive medicine (2 panelists)	
	- Primary care: family medicine (2 panelists), emergency (1 panel)	
Gender	Male	10 (66.7)
	Female	5 (33.3)
Age group (yr)	40s	5 (33.3)
	50s	8 (53.4)
	60s or older	2 (13.3)

**Table 2.** The rating scale of the percent Angoff and the yes/no Angoff methods

Percent Angoff	Yes/no Angoff									
	50%		60%		70%		80%		90%	
	<50%	≥50%	<60%	≥60%	<70%	≥70%	<80%	≥80%	<90%	≥90%
Correct % 0–100	0	1	0	1	0	1	0	1	0	1

**Table 3.** The generalizability model: i×(s:p)

Facet	No.	Content
I (item)	360	-
P (panelist)	15	Internal medicine (2 panelists), pediatrics (2 panelists), psychiatry (2 panelists), surgery (2 panelists), obstetrics & gynecology (2 panelists), family medicine (2 panelists), emergency (1 panel), preventive (2 panelists)
S (specialty)	4	Medicine (internal medicine, pediatrics, psychiatry), surgery (surgery, obstetrics & gynecology), law (preventive medicine), primary care (family medicine, emergency)

**Table 4.** Cutoff scores and failure rates with the PA and YNA methods

Variable	PA %	YNA				
		50% (YNA-50%)	60% (YNA-60%)	70% (YNA-70%)	80% (YNA-80%)	90% (YNA-90%)
No.	360	360	360	360	360	360
Minimum	49.333	0.571	0.357	0.071	0	0
Maximum	82.333	1	1	1	0.857	0.6
Mean	63.536	0.888	0.746	0.475	0.209	0.0399
SD	5.541	0.087	0.132	0.187	0.148	0.07
SE	1.43	2.25	3.41	4.83	3.82	0.02
Mean%						
Cutoff score	**63.5**	88.8	74.6	47.5	20.9	4
Failure rate (%)	**9.1**	99.82	61.26	0.58	0.03	0.00
M-SD						
Cutoff score	**58**	80.1	**61.4**	28.8	6.1	-0.11
Failure rate (%)	**3.49**	88.08	**6.68**	0.03	0.00	0.00
M-SE						
Cutoff score	**62.1**	86.6	71.2	42.7	17.1	0.02
Failure rate (%)	**7.72**	99.42	41.22	0.40	0.03	0.00
M-SEM^a)^						
Cutoff score	**61.5**	86.8	72.6	45.5	18.9	-1.95
Failure rate (%)	**6.68**	99.42	46.15	0.58	0.03	0.00

Hofstee: acceptable cutoff score (56.25%–61.9%) cited by Park et al. [7] in 2020. PA, percent Angoff; YNA, yes/no Angoff; SD, standard deviation; SE, standard error; SEM, standard error of measurement.

a) SEM of observed scores in the Korean Medical Licensing Examination was 1.99.

**Table 5.** Variance component of the generalizability model: i×(s:p)

Standard-setting method	Effect	df	SS	MS	VC	% Variance	G	Phi
Angoff 0–100%	i	359	165,310.9	460.476	22.332	10.4	0.727	0.524
	p	3	168,900.7	56,300.22	29.866	13.9		
	s:p	11	198,642.4	18,058.4	49.857	23.1		
	ip	1,077	132,141.6	122.6942	3.6187	1.7		
	is:p	3,949	433,709.5	109.8277	109.828	51.0		
Y/N Angoff 50%	i	359	37.054	0.103	0.001	1.05	0.153	0.08
	p	3	86.938	28.979	0.017	17.20		
	s:p	11	75.319	6.847	0.019	18.79		
	ip	1,077	88.057	0.082	0.007	7.24		
	is:p	3949	220.865	0.056	0.056	55.72		
Y/N Angoff 60%	i	359	93.12454	0.259	0.008	4.17	0.47	0.327
	p	3	108.2313	36.077	0.017	8.75		
	s:p	11	156.7535	14.250	0.039	20.13		
	ip	1,077	146.3781	0.136	0.002	1.09		
	is:p	3,949	506.8872	0.128	0.128	65.87		
Y/N Angoff 70%	i	359	178.992	0.499	0.022	8.58	0.654	0.543
	p	3	101.864	33.955	0.013	5.07		
	s:p	11	189.829	17.257	0.047	18.43		
	ip	1,077	181.783	0.169	-0.002^a)^	0.00		
	is:p	3,949	690.701	0.175	0.175	67.93		
Y/N Angoff 80%	i	359	105.822	0.295	0.013	7.96	0.645	0.539
	p	3	60.913	20.304	0.005	3.05		
	s:p	11	153.122	13.920	0.038	23.43		
	ip	1,077	108.577	0.101	0.000	0.00		
	is:p	3,949	424.023	0.107	0.107	65.57		
Y/N Angoff 90%	i	359	33.355	0.093	0.004	7.55	0.586	0.524
	p	3	12.180	4.060	0.002	3.14		
	s:p	11	20.691	1.881	0.005	9.35		
	ip	1,077	35.964	0.033	0.000	0.00		
	is:p	3,949	172.231	0.044	0.044	79.96		

I, item; P, panelist; S, specialty; df, degrees of freedom.

a) Negative variance components are set to zero when computing the G coefficient and % of variance.

## Footnotes

**Data availability**
Data files are available from Harvard Dataverse: https://doi.org/10.7910/DVN/D8XT81 Dataset 1. Percent Angoff data (https://doi.org/10.7910/DVN/CBOUPU). Dataset 2. Percent and yes/no Angoff data.

## Supplementary materials

Supplementary files are available from Harvard Dataverse: https://doi.org/10.7910/DVN/D8XT81

Supplement 1. Audio recording of the abstract.
